# Oncological and functional outcomes after testis-sparing surgery in patients with germ cell tumors: a systematic review of 285 cases

**DOI:** 10.1007/s00345-022-04048-6

**Published:** 2022-07-12

**Authors:** Josias Bastian Grogg, Zeynep Hafza Dursun, Joerg Beyer, Daniel Eberli, Cedric Poyet, Thomas Hermanns, Christian Daniel Fankhauser

**Affiliations:** 1 Department of Urology, University of Zurich, Zurich, Switzerland; 2 Department of Oncology, Inselspital Bern, University of Bern, Bern, Switzerland; 3 Department of Urology, Cantonal Hospital of Lucerne, Lucerne, Switzerland

**Keywords:** Urology, Testis cancer, Testis-sparing surgery, Systematic review

## Abstract

**Introduction and objectives:**

In several urogenital cancers, organ-preserving surgery represents the preferred treatment approach, but in patients with testicular germ cell tumors (tGCTs), radical orchiectomy represents the standard of care. This study aimed to summarize published case series assessing oncological and functional outcomes after testis-sparing surgery (TSS) in patients with tGCTs.

**Materials and methods:**

A systematic literature review and individual patient data meta-analysis were conducted of published cases with tGCT treated with TSS.

**Results:**

Of 2,333 reports, we included 32 reports providing data on 285 patients, including 306 testicles treated with TSS. Adjacent germ cell neoplasia in situ (GCNIS) was described in 43%. Hypogonadism and infertility after TSS were diagnosed in 27% and 18%. In patients undergoing adjuvant testicular radiotherapy, hypogonadism was diagnosed in 40%. Patients treated with adjuvant testicular radiotherapy after TSS exhibited a significantly lower incidence of local recurrence (2% vs. 50%, *p* < 0.001). Distant metastases after TSS were observed in 2%.

**Conclusion:**

The current data questions the benefits of TSS in tGCT patients. If at all, TSS should only be offered to well-informed patients with a singular testicle, excellent compliance, a singular tumor less than 2 cm located at the lower pole of the testicle, and normal preoperative endocrine function. Unless patients plan to father a child within a short time frame, adjuvant testicular radiotherapy should be recommended after TSS. Radical orchiectomy remains the standard of care, but future studies may support the use of TSS in selected men.

**Supplementary Information:**

The online version contains supplementary material available at 10.1007/s00345-022-04048-6.

## Introduction

In several urogenital cancers, organ-preserving surgery represents the preferred treatment approach. In men with a testicular germ cell tumors (tGCT), radical orchiectomy is the standard of care. Testis-sparing surgery (TSS) potentially reduces the risk of hypogonadism, testosterone supplementation and infertility but is only a valid treatment option in men with benign or interstitial cell tumors and the risks and benefits of TSS in men with tGCT is unknown. This systematic review and meta-analysis aimed to summarize the published literature of TSS in patients with tGCT.

## Materials and methods

This review was performed according to the Preferred Reporting Items for Systematic Reviews (PRISMA) statement [1, 2]. A literature search of the most important medical electronic databases was conducted to discover relevant articles published until April 25, 2020. Detailed information regarding the data acquisition and data extraction process, our statistical analysis as well as the literature search strategy, are provided in the supplementary file 1.

## Results

### Patient characteristics

Of the 2333 initially identified studies, 384 articles were eligible for full-text review after title and abstract screening. Finally, 32 studies were included, providing data on 285 patients, including 306 testicles treated with TSS (Suppl. Fig. 1).

Overall, the mean age at diagnosis was 31 years (± 5 SD). The median tumor size was 15 mm (IQR: 13–19; Suppl. Table 1). For 277 of 285 (97%) patients, information regarding the histologic subtype of tGCT was provided, of which 171 of 277 (62%) were diagnosed with a pure seminoma, 88 of 277 (32%) with a mixed tGCT, and 18 of 277 (7%) with a pure teratoma. Adjacent germ cell neoplasia in situ (GCNIS) was described in 120 of 277 (43%) specimens. Out of 42 (15%) patients with information on surgical margins, 31 (74%) had negative surgical margins, whereas 11 (26%) cases had positive surgical margins. Data on serum tumor markers were available in 27 of 285 (10%) patients. Out of 27 patients, elevated serum levels of alpha-fetoprotein, beta-human chorionic gonadotropin and lactate dehydrogenase were observed in 4 (15%), 2 (7%), and 1 (5%), respectively.

### Clinical scenarios

**Fig. 1:**
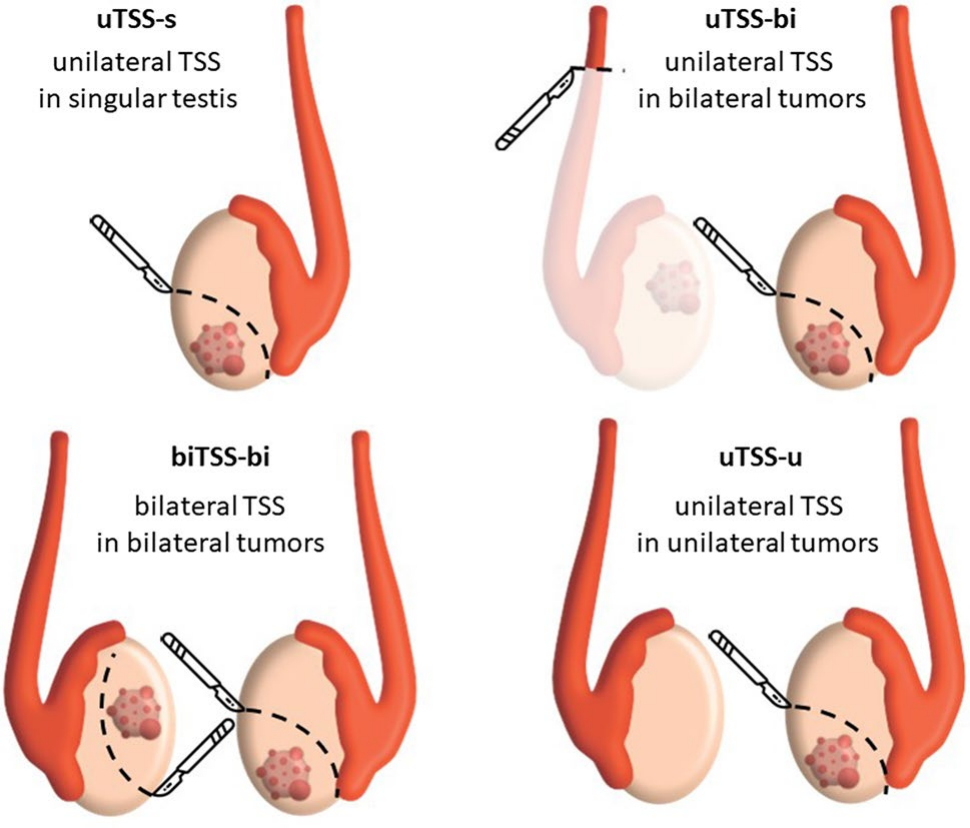
Grouping of patients undergoing TSS for testicular GCT based on the clinical scenario

Four clinical scenarios were defined (Fig. 1). In 119 of 285 (42%) patients, TSS was performed in a singular testis (uTSS-s) due to agenesis, trauma, or orchiectomy more than 24 months before TSS of the contralateral testis. In 51 of 285 (18%) patients, TSS was performed in 1 testicle, whereas the contralateral tumor was treated with orchiectomy (uTSS-bi). In 21 of 285 (7%) cases, synchronous bilateral tumors were treated with bilateral TSS (biTSS-bi). In 14 of 285 (5%) patients, unilateral TSS was used in those with a healthy contralateral testis (uTSS-u). In 80 of 285 (28%) patients, information about the clinical scenario was not clearly described.

### Adjuvant therapy

Adjuvant treatment after TSS was described in 132 of 285 (46%) patients. Adjuvant radiotherapy (RT) at doses of 16 to 20 Gray to the testicles was given in 111 patients. Five patients received radiation to the paraaortic lymph nodes. Adjuvant chemotherapy was used in 26 patients; 11 patients received 2 to 3 cycles of bleomycin, etoposide, and cisplatin (BEP). Four patients received two cycles of carboplatin, and one received four cycles of etoposide–cisplatin (EP). In 9 of 132 (7%) patients, adjuvant retroperitoneal lymph node dissection (RPLND) was performed. Another 12 patients were treated with multimodal adjuvant treatments, including radio/chemotherapy and RPLND (two cases), radiotherapy and chemotherapy (two cases), and radiotherapy and RPLND (three cases).

### Oncological outcomes

#### Local recurrence

Data on local recurrence during follow-up were provided for 270 of 285 (95%) patients. While 236 of 270 (87%) of the patients undergoing TSS exhibited no recurrence after a median follow-up of 38 months (IQR 18–57), 34 (13%) developed local recurrence after a median of 12 months (IQR 8–29; Table 1). The histologies of 34 patients with local recurrence included pure seminoma in 13 (38%), mixed GCT in 6 (18%), pure teratoma in 1 (3%), and was not specified in 14 (41%) patients. Stratified by clinical scenarios, 13 of 119 (11%) had local recurrence after uTSS-s, 1 of 14 (7%) after uTSS-u, 3 of 51 (6%) after uTSS-bi, and 1 of 21 (5%) after biTSS-bi (5%).

**Table 1:** Patient characteristics, treatment and outcome of patients with local and distant recurrence

Author (year)	Nr. of patients	Age	Clinical scenario	Local recurrence free survival (months)	Histology of primary tumour	Size (mm)	GCNIS in TSS testis	Adjuvant treatment after TSS	Positive surgical margin of TSS	Elevated tumour markers at recurrence	Treatment of local recurrence	Response after treatment of local recurrence
Arda et al. [3]	1	28	uTSS-s	24	Mixed GCT	15	0	1/1 Chemo (BEP 2 cycles)	NR	1/1 (AFP, B-HCG)	RO	CR
Bojanic et al. [4]	1	30	uTSS-u	39	Seminoma	12	NR	NR	0/1	NR	RO	CR
Bojanic et al. [5]	1	23	uTSS-s	31	Mixed GCT	20	1/1	1/1 Chemo (NR)	NR	NR	RO	CR
Bojanic et al. [5]	1	20	uTSS-s	60	Seminoma	12	1/1	1/1 None	NR	NR	RO	CR
Bojanic et al. [5]	1	28	uTSS-s	8	Mixed GCT	8	0	1/1 None	NR	NR	TSS	CR
Bojanic et al. [5]	1	30	uTSS-bi	13	Seminoma	8	1/1	1/1 Chemo (NR)	NR	NR	RO	CR
Bojanic et al. [5]	1	16	uTSS-s	12	Seminoma	9	0	1/1 None	NR	NR	TSS	CR
Bojanic et al. [5]	1	35	uTSS-s	13	Seminoma	20	1/1	1/1 None	NR	NR	RO	CR
Bojanic et al. [5]	1	24	uTSS-s	4	Seminoma	14	1/1	1/1 None	NR	NR	RO	CR
Ferretti et al. [6]	1	39	uTSS-bi	6	Seminoma	20	0	NR	0/1	NR	RO	CR
Forbes et al. [7]	1	23	uTSS-s	48	Mixed GCT	7	0	1/1 Chemo (EP 4 cycles)	0/1	1/1 (LDH)	RO	CR
Heidenreich et al. [8]	1	NR	uTSS-bi	9	Seminoma	19	0	None	NR	NR	RO	CR
Heidenreich et al. [9]	4	NR	NR	3, 6, 12, 165	NR	15^a^	3/4	1/4 RT (20Gy), 3/4 none	1/4	NR	RO	3 CR / 1 DOD
Keske et al. [10]	1	NR	uTSS-s	NR	Seminoma	12	1/1	1/1 Chemo (NR) and RT (NR)	NR	0/1	RO	CR
Kizilay et al. [11]	2	NR	NR	22, 28	Seminoma	11^a^	NR	NR	2/2	NR	RO	CR
Lawrentschuk et al. [12]	2	NR	uTSS-s	8, 4	Seminoma	15^a^	2/2	1/2 RT (NR) 1/2 RPLND	0/2	1/2	RO	CR
Nason et al. [13]	10	NR	NR	3	NR	14.1^a^	NR	10/10 None	0/10	1/10	RO	CR
Sener et al. [14]	1	23	biTSS-bi	9	1	8	0	1/1 None	0/1	NR	RO	CR
Steiner et al. [15]	1	NR	uTSS-s	10	Teratoma	17.1^a^	1/1	1/1 None	0/1	NR	TSS	CR
Xiao et al. [16]	1	19	uTSS-s	12	Mixed GCT	22	0	1/1 None	0/1	0/1	RO	CR

Author (year)	Nr. of patients	Age	Clinical scenario	Distant recurrence free survival (months)	Location of distant recurrence	Histology of primary tumour	Size (mm)	GCNIS in TSS testis	Adjuvant treatment after TSS	Positive surgical margin of TSS	Elevated tumour markers at recurrence	Treatment of distant recurrence	Response after treatment of distant recurrence
Forbes et al. [7]	1	23	uTSS-s	52	RP	Mixed GCT	7	0/1	1/1 Chemo (EP: 4 cycles)	0/1	1/1 (LDH)	Chemo (EP: 4 cycles)	CR
Heidenreich et al. [9]	3	NR	Unknown	6, 12, 24	RP, Lung, Mediastinum, Brain	NR	15^a^	NR	NR	NR	NR	2/3 Chemo (BEP: 3 cycles)	2 CR, 1 DOD
Lawrentschuk et al. [12]	1	NR	uTSS-s	4	Micrometastatic disease of unknown origin	Seminoma	15^a^	1/1	1/1 RT (NR)	0/1	1/1 (NR)	1/1 Chemo (BEP: 3 cycles)	CR
Lawrentschuk et al. [12]	1	NR	uTSS-bi	4	RP	Teratoma	11	0/1	1/1 None	0/1	0/1	1/1 Surgery (RPLND)	CR
Morales-Barrera et al. [17]	1	32	uTSS-s	13	RP, Pelvic LN	Seminoma	7	1/1	1/1 None	0/1	NR	RT (NR)	CR
Nason et al. [13]	7	NR	Unknown	NR	LN of unknown location	NR	14.1^a^	NR	0/7 None	0/7	NR	NR	7 CR
Weissbach [18]	1	29	uTSS-bi	24	RP	Mixed GCT	14	1/1	1/1 RT (20 Gy)	NR	NR	Chemo (BEP: 2 cycles), Surgery (RPLND)	CR
Weissbach [18]	1	22	uTSS-s	4	RP	Mixed GCT	12	1/1	1/1 RT (20 Gy)	NR	NR	Chemo (BEP: 4 cycles)	CR

*uTSS-s* unilateral TSS in singular testis (singular testis defined as contralateral testicular agenesis, previous trauma or testicular cancer > 24 months leading to orchiectomy), *uTSS-u* unilateral TSS in unilateral tumor (and healthy contralateral testis), *uTSS-bi* unilateral TSS in bilateral tumor and simultaneous orchiectomy on contralateral testis (or orchiectomy within the last 24 months before TSS), *biTSS-bi* bilateral TSS in bilateral tumor, *NR* not reported, *AFP* alpha-feto protein, *B-hCG* human chorionic gonadotropin, *LDH* Lactatdehydrogenase, *BEP* Bleomycin and Etoposide and Cisplatin, *EP* Etoposide and Cis-Platin, *RT* Radiotherapy, *Gy* Grey, *RO* Radical Orchiectomy, *TSS* Testis Sparing Surgery, *CR* Complete Response, *DOD* Died of disease

^a^Non-individual value

#### Treatment and outcome after local recurrence

Local recurrence was treated with salvage radical orchiectomy in 30 of 34 (88%), of which all patients remained free of disease after a median of 47 months (IQR 36–80). A repeated TSS was performed in two patients without adjuvant therapy for at least 54 and 65 months Bojanic et al. [5]. In the third patient with repeated TSS, a classical seminoma and adjacent GCNIS were diagnosed and adjuvant testicular radiotherapy of 18 Gray was provided after repeated TSS, and the patient remained free of disease Steiner et al. [15]. One patient (3%) with local recurrence did not receive additional treatment due to poor compliance and consequently developed distant metastasis and died of the disease 24 months after initial diagnosis.

#### Risk factors for local recurrence

The mean age at diagnosis was 28 years (± 6 SD) for patients with local recurrence and 31 years (± 6 SD) for patients without local recurrence (*p* = 0.827). The mean tumor sizes were 14 mm (± 5 SD) and 14 mm (± 7 SD) in patients with and without local recurrence, respectively (*p* = 0.945). The analysis regarding the correlation between the presence of GCNIS and local recurrence was performed based on limited individual patient data describing the GCNIS status (70 of 270 patients were available for analysis). In those patients, local recurrence was observed in 6 of 39 (16%) patients with GCNIS versus 7 of 31 (23%) patients without GCNIS (*p* = 0.541).

#### Adjuvant treatment to prevent local recurrence

Individual patient data regarding adjuvant testicular radiotherapy and local recurrence were available in 63 of 270 (23%) patients. Patients treated with adjuvant testicular radiotherapy compared to no adjuvant therapy after TSS showed a significantly lower risk of local recurrence (1 of 43 (2%) vs. 10 of 20 (50%), *p* < 0.001). Individual patient data to study the influence of adjuvant chemotherapy on the risk of local recurrence were available for 47 of 270 (17%) patients. No significant difference regarding local recurrence was observed in patients with or without adjuvant chemotherapy (5 of 17 (29%) vs. 6 of 30 (20%), *p* = 0.493).

#### Distant recurrence

Distant recurrence after TSS was observed in 16 of 285 (2%) patients after a median of 19 months (IQR 9–38; Table 1). The histological diagnosis for 16 patients with distant recurrence included seminoma in 2 (13%), mixed tGCT in 3 (19%) and pure teratoma in 1 (6%). No information on histology was provided in 10 of 16 (63%) cases. In 8 of 16 (50%) patients, metastases were detected in the retroperitoneal lymph nodes (RPLNs). Other locations of metastatic disease in 16 patients included 1 in the chest (6%), 1 in the brain (6%), 1 in the pelvic lymph nodes (LNs) (6%), and 1 in the mediastinal LNs (6%). In 8 of 16 (50%) cases, the exact location of metastatic recurrence was not described (Suppl. Figure 2). After the diagnosis of distant recurrence in these 16 patients, 6 (38%) were treated with chemotherapy (5 patients with 2 to 4 cycles of BEP, 1 with 4 cycles of EP), 2 (13%) received an RPLND, and 1 (6%) patient received radiotherapy to the RPLN and pelvic LN. In 15 of 16 (94%) cases, there was no evidence of disease after systemic treatment during a median follow-up of 52 months (IQR 27–88). One patient with poor compliance developed local and metastatic recurrence and died of the disease 24 months after TSS.

### Functional outcome

Data on functional outcomes were mentioned in 21 studies, and 94 patients were available for individual patient analyses (Table 2). In 37 of 94 (39%) patients, information on gonadal function after TSS was provided. Out of these 37 cases, normogonadism was described in 15 (41%), hypergonadotropic normogonadism in 12 (32%), and hypogonadism in 10 (27%). In patients undergoing adjuvant testicular radiotherapy and available data on functional outcomes, hypogonadism was diagnosed in 8 of 20 (40%) patients. In a subgroup analysis including only patients with TSS in a singular testicle, excluding the subgroup of 8 patients with unilateral TSS with a healthy contralateral testis, hypogonadism was described in 9 of 33 (27%) after TSS and in 7 of 16 (44%) after adjuvant testicular RT.

**Table 2:** Functional outcome for patients after TSS for testicular GCTs

Author (year)	Nr. of patients	Clinical scenario	Age (mean or median)	Follow up time (mean or median months)	Hormonal status after TSS	Androgen substitution needed	Achieved conception	Infertility	Semen analysis after TSS	Symptoms of hypogonadism present	Time of ischemia (min)	Clamping	Adjuvant treatment
Benelli et al. [19]	1	uTSS-bi	25	30	Normogonadism	0/1	NR	NR	NR	NR	16	NR	none
Bojanic et al. [4]	4	uTSS-u	33	60	NR	NR	NR	4/4 preexisting	NR	NR	NR	NR	NR
Bojanic et al. [5]	23	15 pat: uTSS-s 8 pat: uTSS-bi	28	48	NR	NR	4 pat	2/23: preexisting 2/23 pat: new	4/23 Azoospermia	NR	NR	NR	9 pat: chemo (NR)
De Stefani et al. [20]	1	uTSS-u	31	35	Normogonadism	0/1	NR	NR	NR	0/1	18	1/1	none
Demir et al. [21]	1	uTSS-bi	32	30	hypergonadotropic Normogonadism (FSH + , LH +)	0/1	NR	1/1 preexisting	1/1 Azoospermia	0/1	NR	1/1	RT (4680 cGy)
Ferretti et al. [6]	17	uTSS-bi	32	28	NR	NR	NR	8/17: new (unable to father child)	NR	NR	NR	NR	5 pat: chemo (2 pat CAR, 3 pat BEP), 8 pat: RT (16–20 Gy)
Forbes t al. [7]	1	uTSS-s	23	60	preexisting Hypogonadism	1/1 (after TSS)	NR	1/1 Preexisting	NR	NR	NR	NR	Chemo (EP: 4 cycles)
Heidenreich et al. [22]	5	4 pat: uTSS-s 1 pat: uTSS-bi		45	4 pat: hypergonadotropic Normogonadism (FSH + , LH =), 1 pat: Normogonadism	0/5	NR	NR	4/5 Azoospermia	NR	NR	NR	4 pat: RT (20 Gy) 1 pat: chemo (BEP: 2 cycles)
Heidenreich et al. [8]	11	3 pat: uTSS-s 8 pat: uTSS-bi		50	7 pat: hypergonadotropic Normogonadism (FSH + , LH =), 4 pat: Normogonadism	0/11	NR	NR	NR	NR	NR	11/11	4 pat: chemo (3/3: BEP: each 2 cycles, 1/4: PVB: 3 cycles) 7 pat: RT (20 Gy) 1 pat: none
Heidenreich et al. [9]	73	56 pat: uTSS-s 17 pat: biTSS-bi	32	91	NR	11/73	NR	5/10: unable to father child	NR	7 /73	NR	73/73	46 pat: RT (18–20 Gy)
Hughes [23]	1	uTSS-s	20	93	NR	NR	NR	1/1 new	1/1 Azoospermia	0/1	35	1/1	none
Kazem et al. [24]	2	1 pat: uTSS-s 1 pat: uTSS-bi	40	36	NR	0/2	NR	NR	2/2 Azoospermia	0/2	NR	NR	2 pat: RT (1/2: 20 Gy, 1/2: 19.8 Gy)
Keske et al. [10]	3	uTSS-s	30	47.2	2 pat: Normogonadism with low Testosterone	NR	NR	NR	NR	3/3	NR	3/3	1 pat: chemo (NR), RT (NR)
Kirkali et al. [25]	1	uTSS-s	34	50	Normogonadism	0/1	NR	1/1 preexisting	1/1 Azoospermia	NR	NR	1/1	RT (2560 cGy)
Lagabrielle et al. [26]	2	NR	36	20.3	NR	NR	NR	2/2 preexisting	NR	NR	NR	0/2	RT (NR)
Leonhartsberger [27]	7	6 pat: uTSS-bi 1 pat: biTSS-bi	39	52.2	2 pat: Hypogonadism new	0/7	NR	NR	NR	0/7	NR	0/7	NR
Morales-Barrera [17]	2	uTSS-s	28	31.5	NR	NR	NR	2/2 preexisting	2/2 Azoospermia		NR	NR	1 pat: RT (NR)
Sener et al. [14]	1	biTSS-bi	26	18	NR	NR	NR	1/1 preexisting	NR		NR	0/1	NR
Steiner et al. [15]	11	NR	34	59.8	NR	NR	NR	NR	NR		NR	11/11	8 pat: RT (18 Gy)
Weissbach [18]	12	11 pat: uTSS-s 1 pat: uTSS-bi	26	20.5	NR	1/12	NR	NR	NR		NR	12/12	12 pat: RT (20 Gy)
Xiao et al. [16]	6	1 pat: uTSS-s 3 pat: uTSS-u 2 pat: NR	32	56.5	1 pat: Hypogonadism new, 5 pat: Normogonadism	1/6	NR	NR	NR	3 pat: no	NR	NR	1 pat: chemo (BEP: 3 cycles) 4 pat: RT (20 Gy)

*uTSS-s* unilateral TSS in singular testis (singular testis defined as contralateral testicular agenesis, previous trauma or testicular cancer > 24 months leading to orchiectomy), *uTSS-u* unilateral TSS in unilateral tumor (and healthy contralateral testis), *uTSS-bi* unilateral TSS in bilateral tumor and simultaneous orchiectomy on contralateral testis (or orchiectomy within the last 24 months before TSS), *biTSS-bi* bilateral TSS in bilateral tumor, *NR* not reported, *FSH* Follicle-stimulating hormone, *LH* luteinizing hormone, RT Radiotherapy, *Gy* Grey, *CAR* Carboplatin, *BEP* Bleomycin and Etoposide and Cisplatin, *EP* Etoposide and Cis-Platin, *PVB* Cisplatin and Vinblastin and Bleomycin

Information on fertility after TSS was provided in 63 cases out of 94 (67%), and 33 of the 63 (52%) patients were able to father a child after TSS or exhibited preserved fertility in a sperm analysis. In 11 of the 63 (18%) cases, new azoospermia or the inability to father a child was described, whereas infertility was preexisting in 19 of 63 (30%) patients.

## Discussion

In several urogenital cancers, organ-preserving surgery represents a potential or even the preferred treatment approach. For example, in patients with kidney cancer, partial nephrectomy in singular kidneys is a surgical approach which may avoid dialysis. Similarly, in penile cancer, partial penectomy allows preservation of functional and cosmetic favorable results. In contrast, patients with testis or bladder cancer often have a multifocal disease. Further, a complete loss of testicular function has less profound impact on a patient’s daily life compared to for example the loss of renal function. Testis cancer patients will always have the option of preoperative sperm preservation or intraoperative Onco-Testicular Sperm Extraction (ONCO-TESE) as well as testosterone replacement.

In patients with testicular tumors, TSS is currently only accepted as a treatment option in patients with interstitial cell tumors or suspected benign histologies, whereas high inguinal orchiectomy represents the standard of care in patients with tGCT [13, 28, 29]. This systematic review provides further data to discuss the risks and benefits of TSS in patients with tGCT and extends previous systematic reviews in two ways [30–32]. First, we only included tGCT patients to assess the oncological and functional outcomes in this specific group of patients, while previous publications included non-germ cell histologies. Second, pre-processed data of case series were extracted on individual patient level whenever possible, allowing more detailed data analysis and the comparison of different risk and treatment groups.

The provided data support clinicians and patients in balancing oncological risks versus the functional benefits of TSS in selected tGCT patients. The oncological risks of TSS regarding the local and distant recurrence merits further discussion. Previous pathological studies have evaluated orchiectomy specimens and described multifocality in 20% to 30% [33] and adjacent GCNIS in 72% to 90% of patients [9, 34]. Given the risk of 50% for a patient with GCNIS to develop tGCT within 5 years, [35] the risk of local recurrence of at least 25% should be expected after TSS. In this review, we observed a local recurrence risk of only 13% but this lower-than-expected risk could be because of the limited follow-up of the published series and publication bias.

In this analysis, we could not identify any clinical or histopathological risk factors for local recurrence. However, it seems that adjuvant testicular radiotherapy significantly decreases the risk of local recurrence. Therefore, we suggest that adjuvant testicular radiotherapy should be discussed after TSS in patients with testicular GCT unless conception is planned within a few months. In patients planning to father a child in the future, cryopreservation or ONCO-TESE rather than a delay of adjuvant radiotherapy should be considered [36].

Further, as in any man with unilateral testis cancer, we suggest a testicular ultrasound follow-up after TSS for several years. In the case of a local recurrence, the published literature suggests that salvage treatment (redo TSS or orchiectomy) was successful, if early enough, to prevent metastatic disease in most patients. Furthermore, our results reveal a low distant recurrence rate of only 2% compared to the described systemic relapse rates of 15% to 25% for stage I tGCTs treated with radical orchiectomy [37, 38]. This outcome might be because of a selection bias and the corresponding smaller median tumor size in this TSS cohort compared to orchiectomy cohorts.

Critics of adjuvant testicular radiotherapy argue that radiation eradicates testosterone production during long-term follow-up. In this review, 73% of men showed sufficient testosterone production of the remaining testicular tissue, and in even 60% of men after adjuvant testicular RT. However, the assessment of functional endpoints based on published case data is limited due to heterogeneous or missing descriptions regarding the pre- and post-therapeutic gonadal function. Nevertheless, our results are in line with previous data suggesting that normal testosterone levels can be observed in 70% to 100% after 2 to 3 years of follow-up [39] and in 57% after 5 years [40].

### Limitations

Due to the lack of correlated individual patient data in some of the analyzed case series and heterogenic reporting, the presented results have a high risk of bias and should be regarded as hypothesis-generating. Specifically, the limited follow-up data combined with the long-life expectancy in this generally young patient cohort and the possible selection and publication bias might distort these findings. Potentially higher long-term recurrence and hormone deficiency rates may even further tilt the scales in the risk–benefit assessment against the use of TSS. Before more conclusive and applicable recommendations for TSS in tGCT can be made, further research on oncological safety and functional outcome is necessary.

In our opinion, the current data (if at all) justify offering TSS only in well-informed patients with a singular testicle, excellent compliance, a singular tumor less than 2 cm located at the lower pole of the testicle, and normal preoperative endocrine function. Unless patients plan to father a child within a short time frame, adjuvant testicular radiotherapy should be recommended after TSS.

## Supplementary Information

Below is the link to the electronic supplementary material.

Supplementary file1 (DOCX 22 KB)

Supplementary file2 Supplementary File 1 Material and methods, search strategy, disclosures (DOCX 17 KB)

Supplementary file3 Supplementary Figure 1 Flow chart of the study selection process (JPG 105 KB)

Supplementary file4 Supplementary Figure 2 Anatomical locations of metastatic sites both at initial staging and during follow-up (DOCX 244 KB)

## Abbreviations

TSS: Testis-sparing surgery
tGCT: Testicular germ cell tumors
PRISMA: Preferred Reporting Items for Systematic Reviews and Meta-analysis
IQR: Interquartile range
SD: Standard deviation
GCNIS: Germ cell neoplasia in situ
AFP: Alpha-1 fetoprotein
bHCG: Beta human chorionic gonadotropin
LDH: Lactate dehydrogenase
uTSS-s: Unilateral TSS in singular testis
uTSS-bi: Unilateral TSS in bilateral tumors
biTSS-bi: Bilateral TSS in bilateral tumors
uTSS-u: Unilateral TSS in unilateral tumor
RT: Radiotherapy
Gy: Gray
BEP: Bleomycin, etoposide and cisplatin
EP: Etoposide–cisplatin
RPLND: Retroperitoneal lymph node dissection
RPLN: Retroperitoneal lymph nodes
LN: Lymph nodes
