# Supplementary material for: Oncological and functional outcomes after testis-sparing surgery in patients with germ cell tumors: a systematic review of 285 cases
Source: World J Urol. 2022 Jul 12;40(9):2293–303. doi: 10.1007/s00345-022-04048-6 (PMC9427883; doi:10.1007/s00345-022-04048-6)
Supplement: Supplementary file 1 — Supplementary file1 (DOCX 22 KB) [file 345_2022_4048_MOESM1_ESM.docx]

| Table 1: Patient characteristics | | | |  |  |  | |
| --- | --- | --- | --- | --- | --- | --- | --- |
|  | **All** patients (%) | **Group 1 TSS in a singular testis** | **Group 2 Unilateral TSS with healthy contralateral testis** | **Group 3 Unilateral TSS after contralateral orchiectomy** | **Group 4 Bilateral TSS** | **Unknown reason for TSS** | |
| Number of patients | **285** | **119/285 (42)** | **14/285 (5)** | **51/285 (18)** | **21/285 (7)** | **80/285 (28)** | |
| Age (years), Available (%)  Mean (±SD) | 269/285 (94)  31 (± 5) | 112/119 (94)  30 (± 4) | 14/14 (100)  32 (± 7) | 51/51 (100)  31 (± 7) | 21/21 (100)  30 (± 6) | 80/80 (100)  33 (± 7) | |
| Size (mm)  Median (IQR)  Size > 40mm | 282/285 (99)  15 (13 – 19) | 119/119 (100)  15 (14 – 15) | 13/14 (93)  12 (10 – 15) | 49/51 (96)  12 (7 - 19) | **21/21 (100)**  14 (14-15) | **80/80 (100)**  **14 (13-14)** | |
| Histology  Tumor type  Pure seminoma  Mixed GCT  Pure teratoma  GCNIS present  Rete testis invasion  Surgical margins  Negative margins (R0)  Positive margins (R1)  Tumor type contralateral testis  Pure seminoma  Mixed GCT  Pure teratoma | 277/285 (97)  171 (62)  88 (32)  18 (7)  120/140 (43)  17/50 (34)  31/42 (74)  11/42 (26)  29 (11)  18 (7)  6 (2) | 113/119 (95)  83 (74)  42 (37)  12 (11)  88 (78)  15 (13)  7 (6)  3 (3)  21 (19)  12 (11)  2 (2) | 14/14 (100)  8 (57)  5 (36)  1 (7)  0 (0)  0 (0)  13 (93)  0 (0)  NA  NA  NA | 45/51 (88)  27 (60)  14 (31)  4 (9)  22 (49)  2 (4)  7 (9)  1 (2)  14 (31)  13 (29)  4 (9) | **12/21 (57)**    **5 (42)**  **2 (17)**  **1 (8)**    **0 (0)**  **0 (0)**      **6 (50)**  **0 (0)**      **3 (25)**  **1 (8)**  **0 (0)** | **80/80 (100)**  **47 (59)**  **27 (34)**  **11 (14)**  **62 (78)**  **15 (10)**  **3 (4)**  **3 (4)**  **3 (4)**  **1 (1)**  **0 (0)** | |
| Location  Lower pole  Hilus  Upper pole  Peripheral  Focality  Unifocal  Multifocal | 84/285 (30)  22 (26)  3 (4)  47 (56)  1 (1)  7 (8)  4 (5) | 78/119  22 (28)  3 (4)  42 (54)  0 (0)  7 (9)  4 (5) | **-**  **-**  **-**  **-**  **-**  **-** | 13/51 (26)  0 (0)  1 (8)  4 (31)  1 (8)  2 (15)  5 (39) | **-**  **-**  **-**  **-**  **-**  **-** | **54/80 (68)**  **15 (28)**  **0 (0)**  **39 (72)**  **0 (0)**  **-**  **-** | |
| Tumor marker  AFP  Normal  Elevated  bHCG  Normal  Elevated  LDH  Normal  Elevated | 27/285 (10)    23 (85)  4 (15)  25 (93)  2 (7)  20 (95)  1 (5) | 8/113 (7)  8 (100)  0 (0)  8 (100)  0 (0)  3 (38)  1 (13) | 10/14 (71)  10 (100)  0 (0)  10 (100)  0 (0)  10 (100)  0 (0) | 4/51 (8)  1 (25)  3 (75)  2 (50)  2 (50)  2 (50)  0 (0) | **1/21 (5)**  **1 (100)**  **0 (0)**  **0 (0)**  **0 (0)**  **0 (0)**  **0 (0)** | **3/80 (4)**  **2 (67)**  **1 (33)**  **3 (100)**  **0 (0)**  **3 (100)**  **0 (0)** |  |

AFP: alpha-fetoprotein, bHCG: beta-human chorionic gonadotropin, IQR: interquartile range, LDH: Lactate dehydrogenase
SD: Standard Deviation, IQR: Inter Quartile Range,
