# Supplementary material for: Oncological and functional outcomes after testis-sparing surgery in patients with germ cell tumors: a systematic review of 285 cases
Source: World J Urol. 2022 Jul 12;40(9):2293–303. doi: 10.1007/s00345-022-04048-6 (PMC9427883; doi:10.1007/s00345-022-04048-6)
Supplement: Supplementary file 2 — Supplementary file2 Supplementary File 1 Material and methods, search strategy, disclosures (DOCX 17 KB) [file 345_2022_4048_MOESM2_ESM.docx]

**Supplementary file 1**

**Material and Methods**

**Data acquisition and search strategy**

This review was performed according to the Preferred Reporting Items for Systematic Reviews (PRISMA) statement.1, 2 A literature search was conducted using MEDLINE, Embase, Scopus, Cochrane Database of Systematic Reviews, and Web of Science to discover relevant articles published until April 25, 2020. The literature search strategy used a broad approach with several combinations, synonyms, and search terms related to TSS in tGCT and no predefined interventions, controls, or specific outcomes (Supplementary file 1) to capture all relevant publications. Non-English literature was excluded unless the abstract was available in English or the full text was available in French, Spanish, Italian, Turkish, or German. Additionally, the reference lists of the identified publications were screened manually to identify further studies. The detailed search strategy is provided in Appendix 1.

Duplicate articles were filtered using the “close match function” of Endnote and manual de-duplication. Two authors (JG, ZHD) screened the titles and abstracts independently to select publications that fulfilled the eligibility criteria and came to a consensus regarding the inclusion of those studies. Data for the same study that appeared in multiple publications were counted only once in the synthesis. Disagreements were discussed and resolved by consensus or by third-party arbitration (CDF).

**Types of studies, participants, and outcome measures**

We included any case reports or series describing patients with stage I tGCT undergoing TSS. In addition, the studies reporting tGCT recurrence, hypogonadism, testosterone level or fertility were eligible for this review.

Data extraction

A data extraction sheet (based on the Cochrane Consumers and Communication Review Group’s data extraction template) was developed and adapted after pilot testing on 10 randomly selected eligible studies. Data on study design, patient characteristics, clinicopathological risk factors, treatment, follow-up and outcomes were collected. One investigator (ZHD) extracted the data, and another (JG) reviewed the extracted data. Disagreements were discussed and resolved by consensus or by third-party arbitration (CDF). Given that only retrospective case series and reports were identified, we refrained from a study-based meta-analysis, but data were extracted on an individual patient level whenever feasible.

**Statistical analysis**

Descriptive data are presented as the median, interquartile range (IQR), and range. We used weighted medians to estimate the median using the medians of the cohort studies and individual patient ages for single-case presentations. The results for continuous normally distributed variables are expressed as the mean plus or minus the standard deviation (SD) and are compared using Student’s t-tests. Continuous nonnormally distributed variables are presented as the median and IQR and are compared using Wilcoxon rank-sum tests. The results for categorical variables are presented as percentages. All statistical tests were two-sided. A p-value of less than 0.05 was considered significant, and all analyses are considered exploratory and hypothesis-generating.

**Search strategy and terms**

**Title of review**

Oncological and functional outcomes after testis-sparing surgery in patients with germ cell tumors: a systematic review of 285 cases

**Research question**

To assess local and distant recurrence rates as well as fertility and hypogonadism proportions and after partial orchiectomy stratified by tumor size, histology and adjuvant therapies.

**PICO**

| **Population** | Patients with testicular germ cell tumor |
| --- | --- |
| **Intervention** | Partial orchiectomy |
| **Comparison** | Tumor size  Histological subtype (seminoma vs non-seminoma/mixed germ cell tumor)  LVI  Rete testis infiltration  Surgical margin  Different adjuvant therapies, surveillance, chemotherapy, radiation (different doses)  Anatomical location in relation to blood supply (upper, lower pole)  Preoperative hormone status |
| **Outcome** | Local and distant recurrence, fertility, hypogonadism |

Conducted search on April 25th 2020:

**Please note that the three different search term topics are color coded in grey for testicular, yellow for partial orchiectomy**

**Medline (Pubmed)**

(

"Testicular Neoplasms"[Mesh] OR "Seminoma"[Mesh] OR "Neoplasms, Germ Cell and Embryonal"[Mesh] OR

((cancer OR carcinoma OR malignant OR neoplasm OR tumor OR tumour) AND (testicular OR testicle OR testis))

)

**AND**

**(**

**(enucleation OR sparing OR partial OR preserving) AND (organ OR testis OR testicular OR testicle OR orchiectomy OR orchidectomy)**

**)**

| Testis cancer terms | Partial orchiectomy terms | Search results |
| --- | --- | --- |
| X |  | 367,121 |
|  | X | 13,096 |
| X | X | 2057 |

**Embase**

('testis cancer'/exp OR 'non seminomatous germinoma'/exp OR 'seminoma'/exp OR 'germ cell tumor'/exp OR ((testis OR testicular OR testicle) NEAR/3 (cancer OR carcinoma OR neoplasm* OR malignancy or tumor OR tumour)):ab,ti OR nonseminoma*:ab,ti OR 'non seminoma*':ab,ti OR 'non-seminoma*':ab,ti OR seminomatous:ab,ti)

AND

('organ sparing surgery'/exp OR 'testis sparing surgery'/exp OR ((enucleate* OR sparing OR partial OR preserving) NEAR/3(organ OR testis OR testicular OR testicle OR orchiectomy OR orchidectomy)):ab,ti)

| Testis cancer terms | Partial orchiectomy terms | Search results |
| --- | --- | --- |
| X |  | 89.247 |
|  | X | 4,192 |
| X | X | 469 |

**Disclosures**

The authors have nothing to disclose. The results presented in this paper have not been published previously in whole or part.

**Acknowledgments**

None

**Declarations**

Ethics approval and consent to participate

This analysis of published case series does not require a formal ethics approval.

Consent for publication

All authors consented to this publication.

**Availability of data and material**

The datasets used and/or analyzed during the current study are available from the corresponding author on reasonable request.

**Competing interests**

The authors have nothing to disclose.

**Funding**

None
