# Supplementary figures and images for: Oncological and functional outcomes after testis-sparing surgery in patients with germ cell tumors: a systematic review of 285 cases
Source: World J Urol. 2022 Jul 12;40(9):2293–303. doi: 10.1007/s00345-022-04048-6 (PMC9427883; doi:10.1007/s00345-022-04048-6)

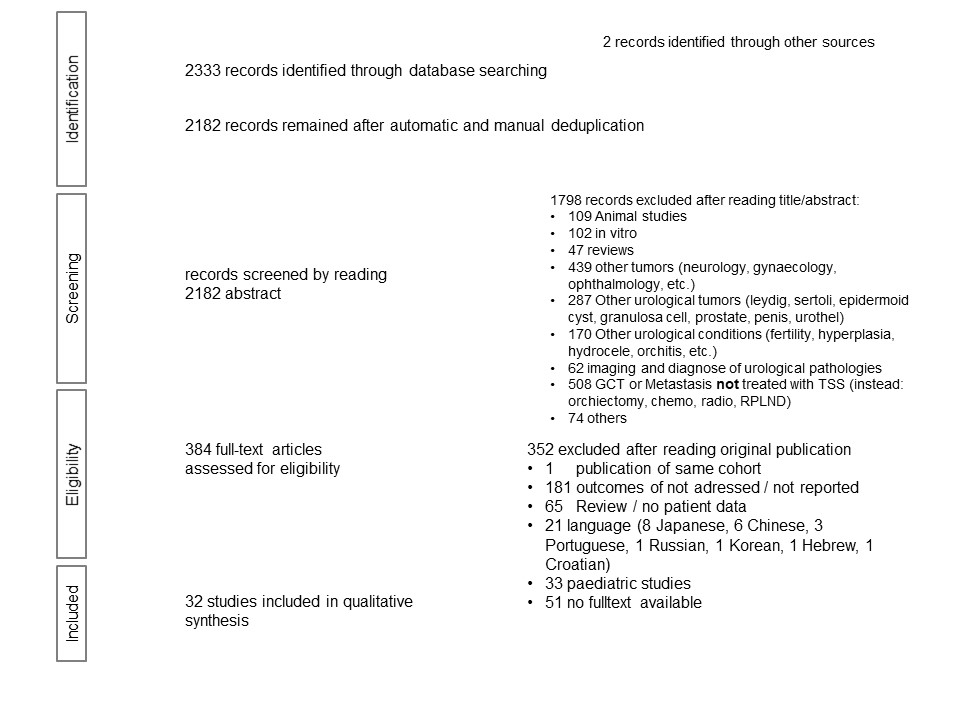

Supplement: Supplementary file 3 — Supplementary file3 Supplementary Figure 1 Flow chart of the study selection process (JPG 105 KB) [file 345_2022_4048_MOESM3_ESM.jpg]
