# Supplementary material for: Oncological and functional outcomes after testis-sparing surgery in patients with germ cell tumors: a systematic review of 285 cases
Source: World J Urol. 2022 Jul 12;40(9):2293–303. doi: 10.1007/s00345-022-04048-6 (PMC9427883; doi:10.1007/s00345-022-04048-6)
Supplement: Supplementary file 4 — Supplementary file4 Supplementary Figure 2 Anatomical locations of metastatic sites both at initial staging and during follow-up (DOCX 244 KB) [file 345_2022_4048_MOESM4_ESM.docx]

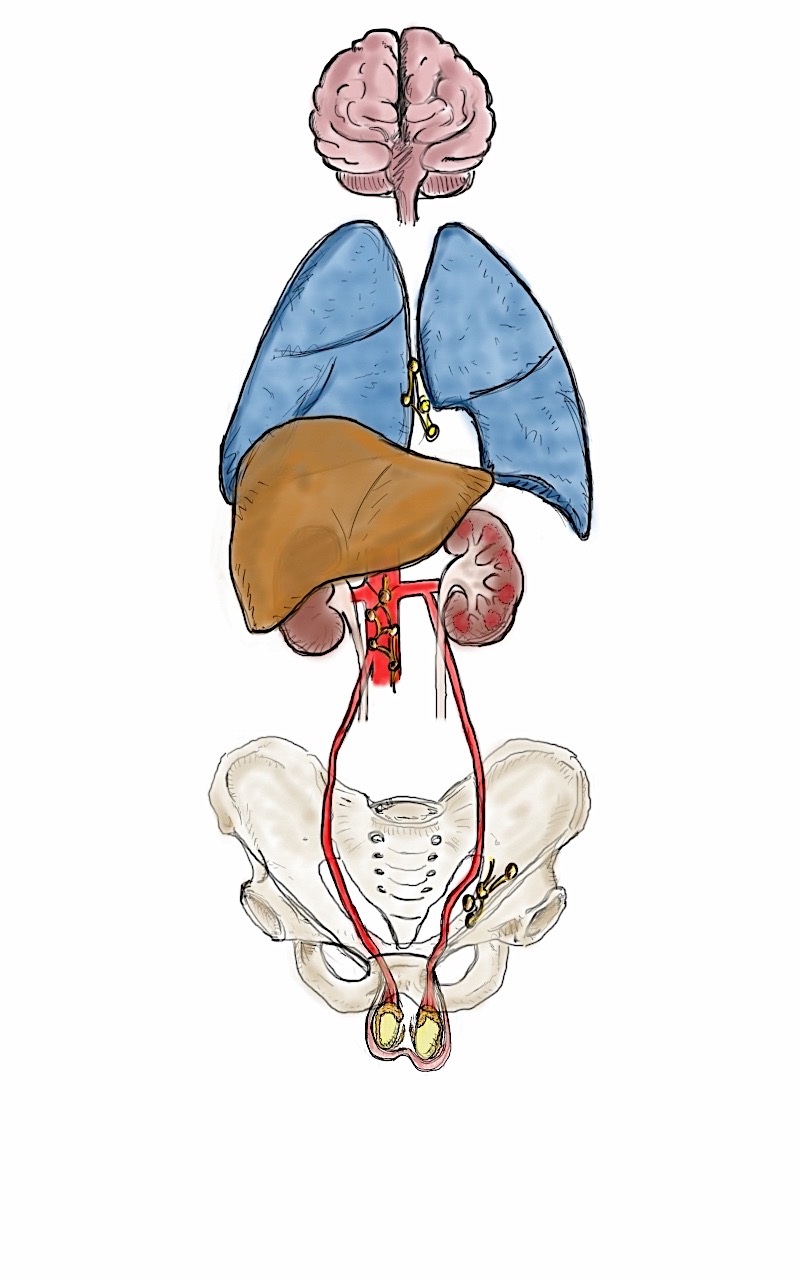


**Unkown location**

8 / 16 (50%)

**pelvic**

**lymph nodes**

1 / 16 (6%)

**mediastinal**

**lymph nodes**

1 / 16 (6%)

**Retroperitoneal**

**lymph nodes**

8 / 16 (50%)

**Lungs**

1 / 16 (6%)

**Brain**

1 / 16 (6%)
